# Paroxysmal Atrial Tachycardia With Atrioventricular Block: A Report of Two Cases

**DOI:** 10.1155/carm/2977958

**Published:** 2026-06-29

**Authors:** Nika Kuridze, Kakhaber Etsadashvili, Saba Tchanturia, Salome Betsuashvili, Tinatin Kavtaradze

**Affiliations:** ^1^ Caucasus Medicine School, Caucasus University, Tbilisi, Georgia, cu.edu.ge; ^2^ Rhythmology Department, G. Chapidze Emergency Cardiology Center, Tbilisi, Georgia; ^3^ AIETI Medical School, David Tvildiani Medical University, Tbilisi, Georgia; ^4^ Outpatient Department, G. Chapidze Emergency Cardiology Center, Tbilisi, Georgia

**Keywords:** AV block, digitalis toxicity, hypokalemia, paroxysmal atrial tachycardia

## Abstract

Paroxysmal atrial tachycardia (PAT) with atrioventricular (AV) block is a rare arrhythmia that can be easily misdiagnosed, yet correct identification is crucial for effective management. This report presents two cases illustrating different etiologies. The first patient, a 78‐year‐old man on digoxin for heart failure, presented with symptoms including tachycardia and dizziness, with elevated digoxin levels confirming toxicity. Following the withdrawal of digoxin and supportive treatment, sinus rhythm was restored without further intervention. The second case involves a 52‐year‐old man with hypertension who developed PAT with AV block and hypokalemia following excessive alcohol intake. Treatment with intravenous potassium normalized his rhythm, and he remained arrhythmia‐free at follow‐up. These cases highlight the importance of identifying underlying causes, such as digitalis toxicity and hypokalemia, in managing PAT with AV block. Accurate diagnosis and individualized therapy are essential for preventing complications and achieving optimal patient outcomes.

**Learning points**
• Paroxysmal atrial tachycardia with AV block can be easily misdiagnosed as atrial fibrillation or flutter; careful ECG interpretation is essential to avoid inappropriate management.
• Always assess for reversible causes such as digoxin toxicity and electrolyte disturbances (especially hypokalemia), as prompt correction can lead to rapid resolution of the arrhythmia without the need for antiarrhythmic therapy.
• This arrhythmia may show transient suppression with adenosine but often recurs unless the underlying cause is corrected—highlighting the importance of addressing the primary trigger rather than relying solely on antiarrhythmic therapy.

## 1. Introduction

Paroxysmal atrial tachycardia (PAT) with atrioventricular (AV) block was first identified more than 100 years ago by Sir Thomas Lewis, who detailed his findings in a 1910 publication [1]. Over the following years, various researchers have shared their findings on this arrhythmia in both healthy individuals and those with heart conditions [2]. Studies by Enselberg et al. have corroborated Luten’s earlier finding that excessive use of digitalis can trigger this arrhythmia [3, 4]. However, PAT with AV block remains relatively rare, often goes unrecognized, and its importance is frequently misunderstood. The exact prevalence of PAT with AV block is not well documented in large‐scale epidemiological studies, making it difficult to provide a precise percentage. However, it is estimated to be quite rare, occurring in less than 1% of patients with arrhythmias. It is commonly confused with atrial fibrillation or flutter, yet the management and progression of these conditions can be quite different, especially when complicated by digitalis toxicity. An accurate diagnosis of PAT with AV block is essential for ensuring appropriate treatment and preventing complications. It guides management decisions and enhances patient safety by avoiding misdiagnosis and improper treatment.

In this report, we present two clinical cases of PAT with AV block, each associated with a different underlying and reversible etiology—digoxin toxicity in one case and hypokalemia in the other. These cases highlight the importance of accurate diagnosis and prompt identification of the precipitating factors, as appropriate correction of the underlying cause is essential for effective management and favorable clinical outcomes. These cases are clinically relevant as they highlight a rare but important arrhythmia that is frequently misinterpreted, potentially leading to inappropriate management and avoidable complications [5].

## 2. Case Presentation

### 2.1. Case 1

**FIGURE 1:**
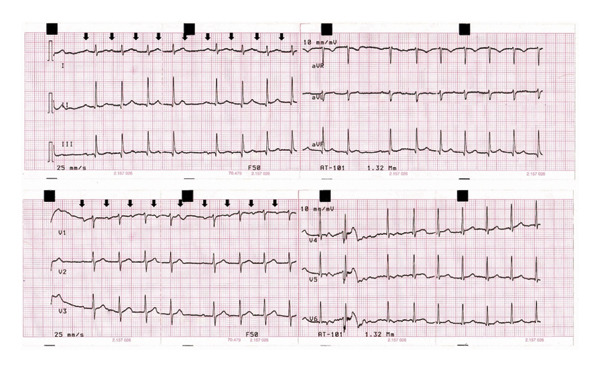
ECG of Case 1 showing paroxysmal atrial tachycardia with AV block. ECG interpretation: atrial rate: 135–142 bpm; ventricular rate: 130 bpm; AV conduction: 1:1 conduction with AV block; P‐wave morphology: normal P wave axis; and presence of isoelectric baseline (paper speed: 25 mm/s, gain: 10 mm/mV, and atrial activity is indicated by arrows).

A 78‐year‐old male presented to the emergency department via ambulance services due to tachycardia. Upon admission, he reported symptoms of general weakness, dizziness, and palpitations. The patient had been receiving long‐term medical therapy for heart failure, including sacubitril/valsartan 26/24 mg b.i.d, bisoprolol 7.5 mg o.d., spironolactone 50 mg o.d., and digoxin 0.25 mg o.d. Notably, the last dose of digoxin was taken 12 h before admission. Clinical measurements revealed a blood pressure of 130/80 mmHg and a heart rate of 130 beats per minute. Electrocardiography indicated atrial tachycardia with an AV block (Figure 1), while echocardiography demonstrated a decreased ejection fraction of the left ventricle (LVEF, 42%).

Standard laboratory evaluation, including renal function parameters (creatinine/eGFR) and electrolyte levels (potassium and magnesium), was within normal limits. Subsequent laboratory investigations highlighted an elevated plasma digoxin concentration of 2.7 ng/mL (therapeutic levels of digoxin: 0.8–2.0 ng/mL). Given these findings, a diagnosis of digoxin‐induced PAT with AV block was established.

Initial management involved carotid sinus massage and intravenous administration of adenosine. As a result, the arrhythmia stopped for a short period.

**FIGURE 2:**
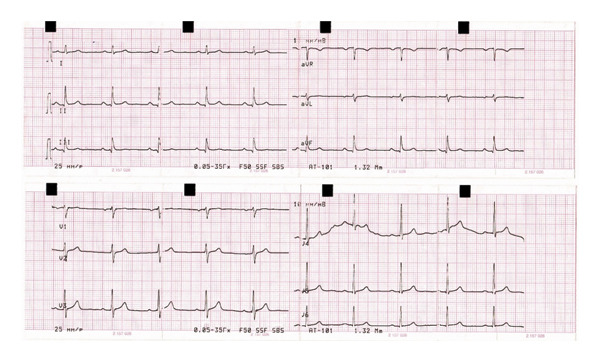
Posttreatment ECG of Case 1 demonstrating restored sinus rhythm (paper speed: 25 mm/s, gain: 10 mm/mV).

In response, digoxin was discontinued, and the patient was administered 700 mL of intravenous 0.9% sodium chloride over 60 min. Additionally, a loop diuretic i.v. (furosemide 40 mg) was given to facilitate diuresis. Administration of the digoxin antidote was not feasible due to its unavailability. Approximately 3 h postintervention, the patient experienced several episodes of atrial tachycardia, which resolved spontaneously, restoring and maintaining sinus rhythm without further antiarrhythmic intervention (Figure 2).

The patient was discharged in stable condition with guideline‐directed therapy for heart failure, and digoxin was permanently withdrawn from the treatment regimen. Follow‐up assessments at 2 weeks and 3 months showed no recurrence of arrhythmias. This finding was confirmed by 24‐h ECG Holter monitoring, which demonstrated a stable cardiac rhythm throughout the follow‐up period.

### 2.2. Case 2

**FIGURE 3:**
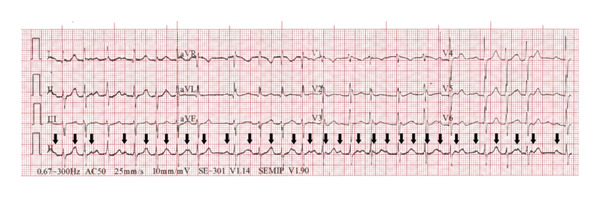
ECG of Case 2 showing paroxysmal atrial tachycardia with AV block. ECG interpretation: atrial rate: 142–250 bpm ; ventricular rate: 115–150 bpm; AV conduction: 1:1/2:1 conduction with AV block; P‐wave morphology: normal P wave axis; and presence of isoelectric baseline (paper speed: 25 mm/s, gain: 10 mm/mV, and atrial activity is indicated by arrows).

A 52‐year‐old male presented to the emergency department experiencing a rapid heart rate and faintness. He reported consuming an unusually high amount of alcohol in the 2 hours before admission, which coincided with the onset of his symptoms. Despite considering himself generally healthy, he has a history of arterial hypertension and is prescribed 10 mg of perindopril and 5 mg of amlodipine daily, with no history of structural heart disease. Upon examination, his heart rate was elevated at 150 beats per minute, and his blood pressure was 160/90 mmHg. Initial tests included an ECG, which revealed PAT with AV block (Figure 3).

**FIGURE 4:**
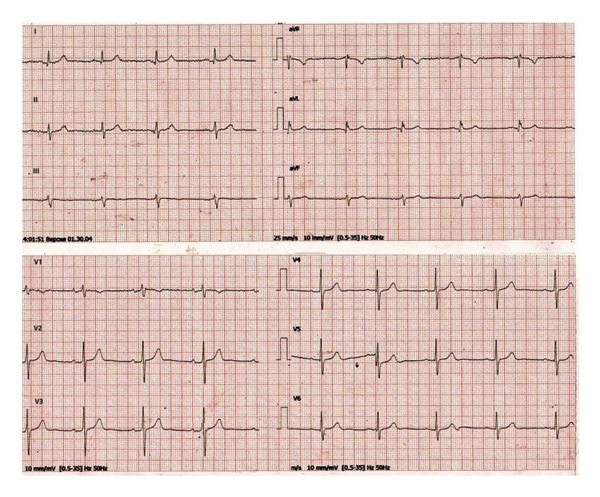
Posttreatment ECG of Case 2 showing sinus rhythm after potassium correction (paper speed: 25 mm/s, gain: 10 mm/mV).

Other diagnostic evaluations, including a cardiac ultrasound and additional lab tests, were normal, except for a low blood potassium level of 3.0 mmol/L (normal levels: 3.5–5.2 mmol/L). The patient’s arrhythmia initially responded to IV adenosine but recurred shortly after. Treatment with intravenous potassium chloride (4%, 100 mL) and Ringer’s solution (500 mL) was initiated to correct hypokalemia. Subsequent monitoring demonstrated normalization of serum potassium levels (3.6 mmol/L) and restoration of sinus rhythm 4 h after admission (Figure 4).

The patient was discharged in satisfactory condition and experienced no further episodes of PAT at follow‐up visits at 1 and 3 months as assessed by 24‐h ECG Holter monitoring.

## 3. Discussion

The cases presented here are typical of PAT with AV block. Both patients presented with symptoms of arrhythmia, including a rapid heart rate and palpitations, which are characteristic of PAT [6]. Electrocardiographic findings were consistent with PAT with AV block. In accordance with current recommendations, both patients received intravenous adenosine [7], leading to the brief termination of the arrhythmia. In the first patient, laboratory evaluation demonstrated elevated plasma digoxin levels; digoxin was discontinued, and supportive management with intravenous diuretics and fluids was initiated. In response, the patient had a few self‐limited episodes of PAT, but his condition was restored, and sinus rhythm was maintained. According to this case, digitalis intoxication appears to be associated with the occurrence of PAT with AV block, but in Case 2, a similar condition was caused by the low level of potassium. After restoring the potassium level, this patient’s response was the same—maintained sinus rhythm without further antiarrhythmic intervention. These two cases of PAT with AV block highlight different underlying causes of the arrhythmia.

PAT with AV block is a well‐recognized manifestation of digoxin toxicity, consistently reported across clinical studies and contemporary expert consensus documents [8, 9]. Current evidence emphasizes that digoxin toxicity may present with a broad spectrum of atrial and AV conduction disturbances, among which PAT with AV block remains a characteristic and clinically important finding. In some cases, digoxin‐induced arrhythmogenesis manifests as PAT accompanied by varying degrees of AV block, often considered a classical electrocardiographic pattern [10]. The underlying pathophysiological mechanisms are multifactorial. Primarily, digoxin inhibits the Na^+^/K^+^‐ATPase pump, resulting in increased intracellular sodium concentration. This promotes enhanced Na^+^/Ca^2+^ exchange, leading to intracellular calcium overload, which facilitates delayed afterdepolarizations and increased atrial automaticity. Additionally, digoxin exerts vagomimetic and sympatholytic effects, contributing to the suppression of AV nodal conduction and the development of AV block. The coexistence of enhanced atrial automaticity and impaired AV conduction explains the typical presentation of PAT with AV block in digoxin toxicity. Several risk factors predispose patients to digoxin‐induced PAT, including elevated serum digoxin concentrations, renal dysfunction—given that digoxin is primarily eliminated via the kidneys—electrolyte disturbances such as hypomagnesemia or hypokalemia, and clinically significant drug interactions. Management of digoxin‐induced PAT requires a comprehensive and targeted approach. Initial treatment involves discontinuation of digoxin and correction of precipitating factors. Rate control may be achieved with appropriate pharmacological agents, while synchronized direct current cardioversion should be reserved for patients with hemodynamic instability. It is important that treatment should primarily focus on the underlying condition contributing to the arrhythmia. Prompt recognition and appropriate management are essential to reduce morbidity and prevent potentially life‐threatening complications [11]. PAT with AV block is classically associated with digoxin toxicity; however, Case 2 highlights hypokalemia as a rare but important alternative etiology. Hypokalemia alters cardiac electrophysiology by reducing extracellular potassium concentration, leading to membrane hyperpolarization, prolonged repolarization, and increased dispersion of refractoriness. These changes promote triggered activity, particularly delayed afterdepolarizations, which can initiate atrial tachycardia [12, 13].

Concurrently, hypokalemia impairs AV nodal conduction by decreasing conduction velocity and increasing nodal refractoriness [14]. This creates a substrate for functional AV block, especially during rapid atrial rates. The combination of enhanced atrial automaticity and depressed AV nodal conduction provides a mechanistic explanation for this uncommon rhythm pattern. Although hypokalemia is well known to cause supraventricular arrhythmias and conduction disturbances, reports of this specific presentation remain limited. Correction of potassium imbalance typically leads to rapid resolution of both tachyarrhythmia and AV block [15].

These two case reports draw attention to the varied etiologies of PAT with AV block, emphasizing the importance of recognizing digitalis toxicity and hypokalemia as significant contributing factors. The first case illustrates a link between digoxin intoxication and PAT with AV block, while the second case underscores the role of hypokalemia in triggering this arrhythmia. These observations highlight the necessity for vigilant rhythm monitoring and tailored management strategies to prevent complications and ensure optimal patient outcomes.

## Author Contributions

Nika Kuridze: conceptualization, methodology, data curation, investigation, project administration, writing–original draft, writing–review and editing, and supervision.

Kakhaber Etsadashvili: conceptualization, methodology, data curation, investigation, project administration, and writing–original draft. Saba Tchanturia: writing–original draft. Salome Betsuashvili: writing–original draft. Tinatin Kavtaradze: writing–review and editing.

## Funding

No funding was received for this manuscript.

## Consent

Written informed consent was obtained from the patients for their anonymized information to be published in this article.

## Conflicts of Interest

The authors declare no conflicts of interest.

## Data Availability

The data that support the findings of this study are available on request from the corresponding author. The data are not publicly available due to privacy or ethical restrictions.
